# Antimicrobial susceptibility profiles of porcine mycoplasmas isolated from samples collected in southern Europe

**DOI:** 10.1186/s12917-020-02512-2

**Published:** 2020-09-03

**Authors:** Rubén S. Rosales, Ana S. Ramírez, María M. Tavío, Carlos Poveda, José B. Poveda

**Affiliations:** Unidad de Epidemiología y Medicina Preventiva, Instituto Universitario de Sanidad Animal y Seguridad Alimentaria, Universidad de Las Palmas de Gran Canaria, C/Trasmontana s/n, 35413 Arucas, Gran Canaria Spain

**Keywords:** Porcine mycoplasmas, Antimicrobial susceptibility, Macrolides, Lincosamides, Pleuromutilins, MIC

## Abstract

**Background:**

*Mycoplasma* (*M.*) *hyopneumoniae*, *M. hyorhinis* and *M. hyosynoviae* are significant pathogens for the porcine industry worldwide. The aim of the present study was to determine the antimicrobial susceptibility of six key antimicrobials (tylosin, tilmicosin, tylvalosin, lincomycin, tiamulin and valnemulin) routinely used for treating infections caused by these pathogens. Twenty-seven *M. hyopneumoniae*, 48 *M. hyorhinis* and 40 *M. hyosynoviae* field strains isolated from clinical samples from different Southern European countries between 2013 and 2018 using broth microdilution method were evaluated.

**Results:**

Tylvalosin exhibited the highest in vitro activity among the macrolides assayed, with MIC_90_ values 4 to 5 two-fold dilutions lower than those of tylosin and tilmicosin. The pleuromutilin valnemulin showed one of the highest in vitro activities against the three mycoplasma species. On the contrary, lincomycin exhibited the highest MIC values of the antimicrobials tested.

**Conclusions:**

The data obtained in the present study supports the use of pleuromutilins and macrolides for the control of infections caused by porcine mycoplasmas. The use of lincomycin for the treatment of porcine mycoplasma infections should be carefully evaluated due to the presence of circulating field isolates with decreased susceptibility to this antimicrobial.

## Background

*Mycoplasma* (*M.*) *hyopneumoniae*, *M. hyorhinis* and *M. hyosynoviae* are considered the most relevant *Mollicutes* to porcine health worldwide, and together with *M. suis*, a non-culturable haemotropic mycoplasma, represent the main pathogenic mycoplasmas of pigs [1, 2]. *M. hyopneumoniae* is a major porcine pathogen, due to its role as the aetiological agent of enzootic pneumonia and also, by interacting with other microorganisms, as a primary pathogen of the porcine respiratory disease complex [3], a disease considered as the most relevant health concern for pig producers [4]. Commercial vaccines are routinely used for the control of this pathogen. However, the analysis of *M. hyopneumoniae* vaccines under field conditions has shown variable efficacy [5], leading in practice to the regular use of antimicrobials against its infections. The antibiotics most frequently used against *M. hyopneumoniae* infections in pigs are aminocyclitols, aminoglycosides, fluoroquinolones, florfenicol, lincosamides, macrolides, pleuromutilins and tetracyclines [6]. On the other hand, *M. hyosynoviae* is one of the main bacterial pathogen involved in pig lameness [7–9]. Infections caused by this pathogen are characterised by a variable progression, leading in most cases to clinical arthritis. Up to date, there are no commercial vaccines available for the control of this microorganism, so the control measures rely on farm management and antimicrobial treatment. Enrofloxacin, lincomycin, tetracyclines, tiamulin and tylosin are commonly used for the treatment of *M. hyosynoviae* infections [10]. Furthermore, *M. hyorhinis* is a ubiquitous porcine pathogen, primarily associated with cases of polyserositis, arthritis and otitis in pigs. In some cases it has been linked to cases of pneumonia, acting as a secondary or opportunistic pathogen. Pneumonia caused by *M. hyorhinis* is clinically indistinguishable from cases produced by *M. hyopneumoniae* [8–10]. Tetracycline, tiamulin, enrofloxacin, tylosin, tilmicosin and lincomycin have demonstrated in vivo efficacy against this microorganism [11, 12]. Commercial vaccines are not available for *M. hyorhinis*, so additional control measures involving improved farm management strategies and the reduction of environmental stressors are required. As described above, the control of porcine mycoplasmosis still depends strongly on the use of antimicrobials. However, the historical overuse of antibiotics in human and animal medicine has led to a current situation of alert, due to the development of resistance that reduces the therapeutic options. In this scenario, the analysis and monitoring of antimicrobial susceptibility has become pivotal in animal health management [13]. The aim of this study was to evaluate the in vitro activity of some of the most relevant antimicrobials used in cases of porcine mycoplasmosis against mycoplasma field strains isolated from Italian, Portuguese and Spanish clinical samples collected between 2013 and 2018.

## Results

### Mycoplasma strains

A total of 27 *M. hyopneumoniae* isolates were obtained from cases of porcine respiratory disease. *M. hyorhinis* isolates (48) were divided into 18 isolates obtained from arthritic joints and 32 from respiratory diseases cases. The 40 *M. hyosynoviae* isolates were obtained from arthritis cases.

### MIC values for *M. hyopneumoniae*

The MIC range, MIC_50_ and MIC_90_ for each antimicrobial tested against *M. hyopneumoniae* are presented in Table 1. For macrolides, isolates tested against tylvalosin showed an MIC range of 0.016–0.06 μg/ml and MIC_50/90_ values of 0.03/0.06 μg/ml. Results obtained for the other two macrolides tested, tylosin and tilmicosin, were higher than those described for tylvalosin, with an MIC range of 0.06–1 and 0.25–1 μg/ml respectively and MIC_50/90_ values at 0.5/1 μg/ml. Lincomycin MIC showed a wide range of dilutions, ranging from 0.06 to 16 μg/ml, including the highest MIC value of all *M. hyopneumoniae* strains tested. MIC_50_ value found for lincomycin was 0.25 μg/ml, while the MIC_90_ value observed was 4 μg/ml. Tiamulin MIC values were similar to those described for tylosin. For technical issues, valnemulin was only tested against 12 Spanish isolates, and it presented the lowest MIC range (0.008–0.03 μg/ml) of all antimicrobial assayed against *M. hyopneumoniae*. The distribution of MIC values per antimicrobials tested can be found in Table 2. Tilmicosin, tylvalosin and valnemulin showed the narrowest distributions, followed by tylosin and tiamulin.

**Table 1:** MIC ranges, MIC_50_ and MIC_90_ values for 27 *Mycoplasma hyopneumoniae* isolates. Results are shown in μg/ml. ^1^Only 12 isolates were tested against valnemulin.^2^MIC_50_ and MIC_90_ values were not calculated due to sample size

		Tylosin	Tilmicosin	Tylvalosin	Lincomycin	Tiamulin	Valnemulin
Spain (22 isolates)^1^	MIC range	0.06–1	0.25–1	0.016–0.06	0.06–16	0.06–0.5	0.008–0.03
	MIC_50_	0.25	0.5	0.03	0.25	0.125	0.008
	MIC_90_	1	1	0.06	4	0.25	0.03
Italy (5 isolates)^2^	MIC range	0.25–0.5	0.5–1	0.016–0.06	0.25–1	0.06–0.5	–
Total (27 isolates)	MIC range	0.06–1	0.25–1	0.016–0.06	0.06–16	0.06–0.5	–
	MIC_50_	0.5	0.5	0.03	0.25	0.125	–
	MIC_90_	1	1	0.06	4	0.5	–
*M. hyopneumoniae* strain J	MIC	0.06	0.25	0.06	0.125	0.125	0.008

**Table 2:** Distribution of 27 *Mycoplasma hyopneumoniae* isolates based on MIC values. Shaded cells correspond to the MIC ranges of each antimicrobial agent

Antimicrobial agent	MIC (μg/ml)															
	0.002	0.004	0.008	0.016	0.032	0.064	0.125	0.25	0.5	1	2	4	8	16	32	64
Tylosin						6		7	10	4						
Tilmicosin								4	11	12						
Tylvalosin				10	7	10										
Lincomycin						6	4	5	5	1		3	2	1		
Tiamulin						8	8	6	5							
Valnemulin			6	3	3											

### MIC values for *M. hyorhinis*

MIC_50_ and MIC_90_ for *M. hyorhinis* isolates and distribution of MIC values are summarised in Tables 3 and 4. MIC range, MIC_50_ and MIC_90_ were comparable for tylosin and tilmicosin, with just a 2-fold dilution difference in the lowest MIC value and MIC_50_ of tylosin. Tylvalosin showed a reproducible pattern of MIC distribution between the three countries of origin of the isolates, displaying one of the lowest MIC_50_/MIC_90_ of the antimicrobials tested (0.016/0.125 μg/ml). MIC values for lincomycin displayed the highest MIC values for all the countries and antimicrobials studied, with an MIC range of 0.125- > 64, and the highest MIC_90_, with a value of 16 μg/ml. MIC range for tiamulin was comparable to the range observed for tylosin (MIC range 0.06–8 μg/ml). Valnemulin MIC values were comparable to tylvalosin for this mycoplasma, apart from MIC_90_ value, that was the lowest observed for *M. hyorhinis* (0.03 μg/ml). The MIC distribution for all antimicrobials tested showed a broader distribution in comparison to those described for *M. hyopneumoniae*, with lincomycin presenting the widest distribution observed.

**Table 3:** MIC ranges, MIC_50_ and MIC_90_ values for 48 *Mycoplasma hyorhinis* isolates. Results are shown in μg/ml. ^1^Only 19 isolates were tested against valnemulin. ^2^Only 8 isolates were tested against valnemulin. ^3^A total of 27 isolates were tested against valnemulin

		Tylosin	Tilmicosin	Tylvalosin	Lincomycin	Tiamulin	Valnemulin
Spain (25 isolates)^1^	MIC range	0.125–0.5	0.25–2	0.016–0.06	0.125–32	0.06–0.5	0.008–1
	MIC_50_	0.25	1	0.016	0.5	0.125	0.016
	MIC_90_	0.5	1	0.03	1	0.25	0.125
Italy (13 isolates)	MIC range	0.25–8	0.5–8	0.016–0.5	0.125- > 64	0.125–8	–
	MIC_50_	2	0.5	0.125	4	0.25	–
	MIC_90_	4	4	0.5	32	8	–
Portugal (10 isolates)^2^	MIC range	0.125–2	0.5–2	0.016–0.25	0.125–16	0.06–2	0.008–0.03
	MIC_50_	0.125	1	0.016	0.5	0.125	0.016
	MIC_90_	1	2	0.125	8	2	0.016
Total (48 isolates)^3^	MIC range	0.125–8	0.25–8	0.016–0.5	0.125- > 64	0.06–8	0.008–1
	MIC_50_	0.25	0.5	0.016	0.5	0.125	0.016
	MIC_90_	2	2	0.125	16	0.5	0.03
*M. hyorhinis* strain BTS-7	MIC	0.06	0.25	0.016	0.125	0.03	0.016

**Table 4:** Distribution of 48 *Mycoplasma hyorhinis* isolates based on MIC values. Shaded cells correspond to the MIC ranges of each antimicrobial agent

Antimicrobial agent	MIC (μg/ml)																
	0.002	0.004	0.008	0.016	0.032	0.064	0.125	0.25	0.5	1	2	4	8	16	32	64	> 64
Tylosin							14	14	11	1	3	4	1				
Tilmicosin								6	17	17	4	3	1				
Tylvalosin				26	7	6	5	2	2								
Lincomycin							7	6	19	5		1	2	4	3		1
Tiamulin						12	20	9	2		2	1	2				
Valnemulin			7	12	5		1			2							

### MIC values for *M. hyosynoviae*

MIC ranges, MIC_50_ and MIC_90_ for *M. hyosynoviae* were fairly similar between countries, with just one 2-fold dilution difference between MIC values and the geographical origin of the samples (Tables 5 and 6). Valnemulin and tylvalosin displayed the lowest MIC_50_/MIC_90_ for this pathogen (0.016/0.06 μg/ml). The highest MIC_50_/MIC_90_ for *M. hyosynoviae* were those observed for tilmicosin, with concentrations of 1 and 2 μg/ml respectively.

**Table 5:** MIC ranges, MIC_50_ and MIC_90_ values for 40 *Mycoplasma hyosynoviae* isolates. Results are shown in μg/ml. ^1^Only 12 isolates were tested against valnemulin. ^2^Only 2 isolates were tested against valnemulin. ^3^MIC_50_ and MIC_90_ values were not calculated due to sample size. ^4^A total of 14 isolates were tested against valnemulin

		Tylosin	Tilmicosin	Tylvalosin	Lincomycin	Tiamulin	Valnemulin
Spain (18 isolates)^1^	MIC range	0.25–1	0.5–2	0.008–0.06	0.25–1	0.03–0.25	0.016–0.06
	MIC_50_	0.5	1	0.016	0.25	0.06	0.016
	MIC_90_	1	1	0.06	0.5	0.25	0.06
Italy (18 isolates)	MIC range	0.5–1	0.5–2	0.008–0.06	0.25–1	0.03–0.5	–
	MIC_50_	0.5	1	0.016	0.5	0.5	–
	MIC_90_	1	2	0.016	1	0.5	–
Portugal (4 isolates)^2,3^	MIC range	0.5–1	0.5–1	0.016	0.25–1	0.06–0.5	0.016–0.06
Total (40 isolates)^3^	MIC range	0.25–1	0.5–2	0.008–0.06	0.25–1	0.03–0.5	0.016–0.06
	MIC_50_	0.5	1	0.016	0.5	0.125	0.016
	MIC_90_	1	2	0.06	1	0.5	0.06
*M. hyosynoviae* strain S16	MIC	0.25	0.25	0.016	0.125	0.03	0.008

**Table 6:** Distribution of 40 *Mycoplasma hyosynoviae* isolates based on MIC values. Shaded cells correspond to the MIC ranges of each antimicrobial agent

Antimicrobial agent	MIC (μg/ml)																
	0.002	0.004	0.008	0.016	0.032	0.064	0.125	0.25	0.5	1	2	4	8	16	32	64	> 64
Tylosin								4	25	11							
Tilmicosin									13	20	7						
Tylvalosin			13	20		7											
Lincomycin								13	16	11							
Tiamulin					3	10	5	5	17								
Valnemulin				7		7											

## Discussion

This investigation was aimed at evaluating the antimicrobial susceptibility patterns of selected members of the macrolide, lincosamide and pleuromutilin antimicrobial families against isolates of the most clinically relevant porcine mycoplasmas collected from Southern European countries. Macrolides, lincosamides and pleuromutilins are extremely relevant for porcine health. For instance, the World Organisation for Animal Health (OIE) classifies these antimicrobial families as either critically (macrolides) or highly important antimicrobials (lincosamides and pleuromutilins) in veterinary medicine [14]. Furthermore, these antimicrobials have remained pivotal for the treatment of infections caused by porcine mycoplasmas in the last decades [3, 12, 15, 16]. Due to the limited number of isolates analysed per country, a valid comparison of the MIC values based on their geographical origin could not be achieved.

In our study tylvalosin exhibited the highest in vitro activity among the macrolides assayed against the three mycoplasma species, with MIC_90_ values 16 to 32-times lower than those of tylosin and tilmicosin. Similar results were obtained by Tavío et al. [17] after evaluating field strains of *M. hyopneumoniae*, as well as for the avian mycoplasma *M. synoviae* and *Mycoplasma* sp. 1220 [18, 19]. Freely accessible tylvalosin MIC values for mycoplasmas are limited up to date to *M. hyopneumoniae*, *M. hyorhinis*, *M. synoviae*, *Mycoplasma* sp. 1220 and *M. gallisepticum* [17–20]. However, the MIC values observed in our work for this antimicrobial against *M. hyorhinis* and *M. hyosynoviae*, and the in vitro [17] and in vivo efficacy [21] demonstrated for the treatment of *M. hyopneumoniae* infections, gives prominence to tylvalosin as one of the most effective macrolides against porcine mycoplasmosis, together with tulathromycin, a 15-membered macrolide. Up to date, there are limited comparative data between tylvalosin and tulathromycin MIC values for porcine mycoplasmas. Felde et al. [22] described tulathromycin MIC_90_ values of 1 and 4 μg/ml, 1–4 two-fold dilutions higher to those observed for tylvalosin after analysing Central European *M. hyopneumoniae* isolates. Conversely, Klein et al. [23] described MIC_90_ values for tulathromycin of 0.004 μg/ml, after analysing 50 European *M. hyopneumoniae* isolates, however, the authors did not include tylvalosin in their analysis. In addition, decreased susceptibility to tylosin and tilmicosin has been previously described in European *M. hyosynoviae* and *M. hyopneumoniae* isolates [10, 24]. Although tylvalosin was not evaluated in these studies decreased susceptibility for this antimicrobial has yet to be described. The relatively recent application of tylvalosin for therapy purposes in pigs may explain the absence of decreased susceptibility for this antimicrobial. Also, Andersen and others [25], described the presence of alternative bacterial resistance mechanisms for tylosin in comparison to tylvalosin, which can explain the differences in MIC values between members of the 16-membered ring class of macrolides. The tylosin and tilmicosin MIC values were in agreement with those found in *M. hyosynoviae* [10], *M. hyorhinis* [12] and *M. hyopneumoniae* [17, 23]. Based on our data, the macrolides could still be utilised as first-line antimicrobial treatment for the control of mycoplasma disease in pigs. However, evidence about the emergence of macrolide-resistant *M. hyopneumoniae* strains [17, 22, 24, 26], suggests the need for a rational use of this group of antimicrobials in the porcine industry.

With regards to lincomycin, this antimicrobial exhibited the highest MIC values of all the antimicrobial tested, with one strain of *M. hyorhinis* presenting a MIC value of > 64 μg/ml. Lincomycin MIC values for *M. hyopneumoniae* displayed a decreased susceptibility pattern in comparison to Spanish isolates [17], evidenced by 32 times higher MIC_90_ values. However, similar values were observed in previous studies [24, 26]. Current strategies for the control of *M. hyopneumoniae* include the respiratory exposure of gilts to lung homogenates containing viable strains of this pathogen [27]. However, the presence of circulating strains with decreased susceptibility to certain antimicrobials, as found in our work, or even harbouring multidrug resistance phenotypes [26], requires a careful evaluation of the antimicrobial susceptibility patterns of the strains included in the homogenate prior administration to gilts, in order to reduce the dissemination of resistant isolates of *M. hyopneumoniae* in the farm. Lincomycin has been previously described as effective in vitro against *M. hyorhinis* [12, 28]. Conversely, our data suggest the presence of resistant strains of *M. hyorhinis* against this antimicrobial in the population studied, in agreement with the data published by Bekö et al. [20] in Hungarian isolates of this pathogen. *M. hyorhinis* is a common commensal of the upper respiratory tract of pigs, acting as an opportunist pathogen of immunocompromised animal in a variety of clinical presentation [10]. In our study, the population *M. hyorhinis* analysed showed a marked decrease in susceptibility for lincomycin. This antimicrobial has been extensively used as part of in-feed medication in porcine farming, administered orally in premix, oral powder and oral solution for years [29]. Therefore, it can be hypothesised that the intensive selective pressure due to in-feed medication for long periods has facilitated the development of a marked decreased susceptibility in our *M. hyorhinis* strain population. This selective pressure may have enabled in the same way the lincomycin susceptibility and MIC value distribution observed in the *M. hyosynoviae* and *M. hyopneumoniae* population evaluated. *M. hyosynoviae* presented similar MIC values to previous reports against lincomycin, with little to no variation observed [10].

Differences in effectivity between pleuromutilins were observed in the three mycoplasma species analysed as previously described [17], with valnemulin exhibiting 8 to 16 times higher activity than tiamulin. MIC values for tiamulin and valnemulin against *M. hyopneumoniae* were in agreement with those previously reported by Tavío et al. [17], and similar to the MIC values observed in isolates from Central Europe, where all isolates were susceptible to a concentration equal or lower than 0.039 μg/ml [22]. However, Klein et al. [23], reported lower MIC values for 50 European isolates of *M. hyopneumoniae*, with a difference in MIC_90_ found between studies of 8 to 16 times. Also, Hannan et al. [16] described lower MIC values for *M. hyopneumoniae* and *M. hyosynoviae* against both pleuromutilins. These data suggest a potential decreased activity against pleuromutilins for these two porcine pathogens. *M. hyorhinis* MIC data presented the broadest range of values for both pleuromutilins tested, in conjunction with the highest MIC values (8 μg/ml for tiamulin and 1 μg/ml for valnemulin). Other authors have described lower MIC values for both pleuromutilins in *M. hyorhinis* for Hungarian isolates [18], with similar results to those described for *M. hyopneumoniae* in the same region after testing the same antimicrobials [22]. Reduced susceptibility to pleuromutilins in mycoplasmas has been linked to mutations in the 23S rRNA gene [30], mutations that also conferred cross-resistance to lincomycin, tilmicosin and tylosin. Even though there are no described mechanisms of pleuromutilin resistance in *M. hyorhinis*, macrolide and lincomycin decreased susceptibility has been associated to multiple mutations in the same gene [31], suggesting a possible cross-resistance phenomenon between pleuromutilins, lincomycin, tilmicosin and tylosin based on the high MIC values for these antimicrobials observed in the *M. hyorhinis* population studied.

## Conclusions

In conclusion, among the tested antimicrobials, valnemulin and tylvalosin displayed the greatest in vitro activity against the three porcine mycoplasmas species collected from Southern European countries. Although active for *M. hyosynoviae*, the high MIC values observed for lincomycin against *M. hyorhinis* and *M. hyopneumoniae* isolates, suggest the need for a more rational approach to the use of this antimicrobial in cases of porcine mycoplasmosis. The molecular basis of the potential cross-resistance for lincomycin, tilmicosin, tylosin and both pleuromutilins in *M. hyorhinis* should be analysed. The differences observed between isolates from different European regions draw special attention to the need for standardised antimicrobial susceptibility testing. Besides, coordinated monitoring schemes for these pathogens in Europe are essentialto effectively tackle the potential emergence of resistant mycoplasma strains in order to maintain an optimal level of health and welfare in the porcine industry.

## Methods

### Mycoplasma strains

Strains were isolated from clinical samples submitted by field veterinarians for routine diagnosis to the mycoplasma diagnostic service of the Instituto Universitario de Sanidad Animal y Seguridad Alimentaria (Gran Canaria, Spain) between 2013 and 2018. Samples were sourced from farrow-to-finish farms and obtained from fattening pigs, gilts and sows with no previous antimicrobial treatment. Clinical samples analysed included nasal swabs from those animals with respiratory disease symptoms compatible with mycoplasmal pneumonia and synovial fluid from cases of lameness.

The isolation of the strains was performed as previously described [32], using modified Friis broth supplemented with equal parts of horse and porcine serum [17]. Pure cultures were obtained as previously described [32, 33] and the identity confirmed by biochemical characteristics and specific PCR testing [3, 34]. Strain distribution by species and country of origin can be found in Tables 1, 3 and 5.

The type strains of *M. hyopneumoniae* (J), *M. hyorhinis* (BTS-7) and *M. hyosynoviae* (S16) were used as a positive control. All control strains were sourced from the National Collection of Type Cultures (NCTC).

### Antimicrobials

Tiamulin, tilmicosin, valnemulin and lincomycin were obtained from Fluka Analytical (St Louis, Missouri, USA). Tylosin was obtained from Serva (Heidelberg, Germany) and tylvalosin from Eco Animal Health (London, UK). All antibiotic stock solutions were sterilised by filtration through 0.2 μm pore size membrane filters (Millipore).

### Minimum inhibitory concentration testing

Minimum inhibitory concentration (MIC) testing was performed using a microbroth dilution method as previously described [35], using 96-well round base polystyrene microtitre plates (Sarstedt, Nümbrecht, Germany). Antimicrobials were added into the wells following a doubling dilution pattern in order to obtain a final concentration of antibiotic per well that ranged from 0.002 to 64 μg/ml. 100 μl of each antimicrobial dilution was added into each well and inoculated with 100 μl of a 48-h mycoplasma culture. The bacterial load present in each inoculum was calculated as described before [17], and adjusted to a final concentration of 10^5^ colour-changing units/ml of mycoplasma per well. Inoculated plates were then incubated at 37 °C with constant shaking at 150 rpm in a humidified atmosphere until growth in the drug-free control wells was evident. Bacterial growth was examined daily until a colour change was observed for a maximum of 21 days. MIC testing was performed on three different days, and duplicates of each strain were performed on each of the testing days. MIC was defined as the lowest concentration that completely inhibited growth, shown by a lack of colour change at the time that the drug-free growth control exhibited a colour change, while the negative control remained unchanged. MIC_50_ and MIC_90_ are defined as the lowest concentration that completely inhibited growth in 50 and 90% of the population studied respectively, and MIC ranges were also calculated. Due to the lack of official breakpoints for porcine mycoplasmas, the percentage of resistant isolates was not calculated. MIC values of the type strains can be found in Tables 1, 3 and 5. A total of five independent observations per type strain was performed.

## Data Availability

The datasets used and/or analysed during the current study are available from the corresponding author on reasonable request.

## Abbrevations

MIC: Minimum inhibitory concentration
NCTC: National Collection of Type Cultures
OIE: World Organisation for Animal Health
μl: Microlitre
μg/ml: Microgram/millilitre
